# Case of Partial Accommodative Esotropia With Well-Developed Binocular Function After Frequent Interventions

**DOI:** 10.7759/cureus.117222

**Published:** 2026-09-30

**Authors:** Yuki Ohara, Takashi Negishi, Ayano Sakamoto, Sumie Hisatake

**Affiliations:** 1 Department of Ophthalmology, Juntendo Shizuoka Hospital, Shizuoka, JPN; 2 Department of Ophthalmology, Juntendo University, Tokyo, JPN; 3 Department of Ophthalmology, Juntendo Tokyo Koto Geriatric Medical Center, Tokyo, JPN

**Keywords:** accommodative esotropia, binocular function, partially accommodative esotropia, prism therapy, strabismic amblyopia

## Abstract

Esotropia in children significantly impacts visual development, particularly if occurring in infancy, where it poses a high risk of amblyopia and disrupts binocular function acquisition. We report the progressive management of a boy who developed esotropia at the age of two years and one month. Initial assessment revealed hyperopia, managed with spectacles and occlusion therapy for amblyopia. Over time, the esotropia evolved into partially accommodative esotropia, necessitating frequent prescription adjustments of glasses with Fresnel prisms and surgical intervention. It resulted in the maintenance of good visual function and stereopsis. This case underscores the importance of early detection and continuous, tailored interventions in managing accommodative esotropia to prevent amblyopia and promote binocular vision. Through regular follow-up and adaptive treatment strategies, the patient achieved and maintained favorable visual outcomes until at least the age of 12 years, demonstrating the effectiveness of a comprehensive, long-term approach to managing complex esotropia.

## Introduction

Esotropia in children can have a significant impact on visual development [1]. Particularly in infants, the onset of esotropia interferes with the acquisition of binocular function and carries a high risk of causing amblyopia-a condition of reduced visual acuity in one or both eyes due to abnormal visual experience during critical developmental periods [2,3]. In the treatment of esotropia, early detection and early intervention are important, and appropriate case-specific treatment options such as spectacle fitting, occlusion training, prism therapy, and surgery are required [4,5]. Among the subtypes, accommodative esotropia is relatively well recognized, and its ocular alignment generally improves with hyperopic correction [6,7]. However, a proportion of patients progress to partially accommodative esotropia, a condition in which full hyperopic correction improves but does not completely eliminate the deviation, requiring additional therapeutic interventions [7]. Management of these cases can be challenging due to fluctuating deviation, risk of binocular disruption, and the need for long-term monitoring. These cases often require additional interventions, including prisms [8].

In addition to visual acuity, the acquisition and long-term maintenance of binocular function and stereopsis are critical outcome measures in the evaluation of treatment efficacy [9]. However, longitudinal clinical data describing the natural course and visual outcomes of partially accommodative esotropia remain limited in the literature, particularly regarding stereopsis preservation over extended follow-up. Understanding how different therapeutic modalities can be optimally combined in such cases is therefore of considerable clinical importance. In this context, we report a case of a boy with accommodative esotropia who later developed a partially accommodative type but achieved and maintained good binocular function, including high-grade stereopsis, through a series of tailored, long-term interventions extending over 10 years. This case illustrates the practical value of continuous adaptive management in preserving binocular visual outcomes despite disease progression and multiple treatment modalities.

## Case presentation

Throughout this report, prism diopters are denoted as Δ, esotropia as ET, esophoria as EP, and intermittent esotropia as ET'. Measurements were performed using the prism alternate cover test at distance (6 m) and near (33 cm) unless otherwise specified. A boy aged two years and one month presented to the clinic with a one-month history of esotropia. At the initial examination, the angle of deviation, measured using the prism alternate cover test, was 14 prism diopters (Δ) of esotropia (ET) at distance (6 m) and 8 Δ at near (33 cm) fixation without optical correction. No abnormal head posture, including chin elevation or head tilt, was observed at presentation. Refraction using 1% atropine sulfate ophthalmic solution revealed hyperopia of +4.0 D in the right eye and +4.5 D in the left eye, and he started wearing glasses with the same power. The esotropia was initially attributed to uncorrected hyperopia, consistent with accommodative esotropia. Assessment of binocular single vision (BSV) at this early age was limited because of the patient's young age and limited cooperation. Infantile esotropia was excluded based on the later age of onset (2 years) and the presence of significant hyperopia. The initial improvement with hyperopic correction confirmed the accommodative component. At the age of two years and seven months, he showed an aversion reaction in his left eye, indicating amblyopia, and started full-time right eye occlusion (except one day per week) to promote visual development in the left eye. At three years and three months, the prescription for glasses was changed, and at three years and four months, the patient showed worsening esotropia with a residual deviation of approximately 25 Δ at distance despite full hyperopic correction, indicating progression to partially accommodative esotropia. Alternating fixation was observed, and a 20Δ base-out Fresnel prism was placed on the right eye to maintain fusion and promote binocular function. The prism was prescribed for full-time wear to maintain fusion and promote binocular function development. The glasses were made again at four years and three months with the same power. At five years and four months, his visual acuity was (1.2×+4.50 D cyl+0.50 D Ax 90°) in the right eye and (1.2×+5.00 D cyl+1.00 D Ax 90°) in the left eye. Good ocular alignment and stereopsis were achieved while wearing the same glasses. Later, at the age of five years and 11 months, the patient's right eye showed decreased visual acuity, so the membrane prism was changed to 10Δ base-out in both eyes for bilateral application with full-time wear, and the left eye started to be occluded. At six years and two months, residual esotropia was still observed during follow-up (Figure 1).

**Figure 1:**
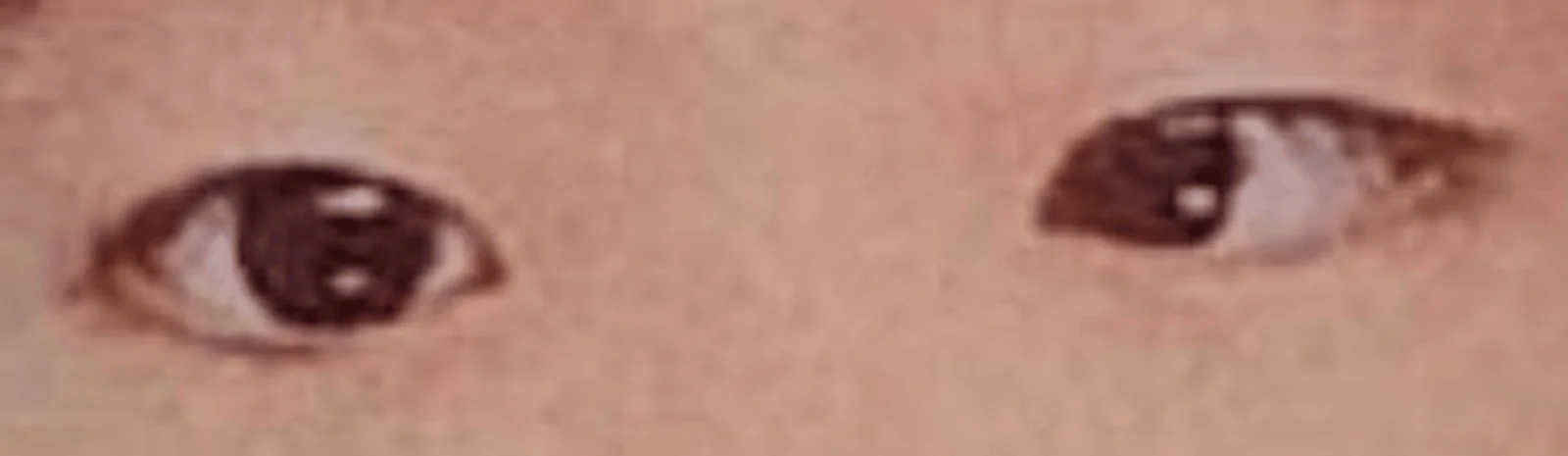
Image at six years and two months. Esotropia was observed in the left eye.

At seven years and four months, a face turn had developed, which was considered to be caused by mild restriction of abduction, and he underwent a 4 mm resection of the lateral rectus muscle of the left eye. The Fresnel membrane prism was discontinued, and his postoperative deviation decreased to 16Δ EP' at distance and 14Δ EP at near with full hyperopic correction. Thereafter, at eight years and one month, stereopsis could not be confirmed in the fly section of the Stereo Fly test, but it was determined that the patient was looking above the frame of his glasses. After adjusting the fitting of the glasses, the patient's ocular position and stereopsis improved, and the number of correct answers was 4 out of 9 in the *circles* section of the Stereo Fly test (140 seconds of arc). The prescription for glasses was changed once a year until the child was 5 years old, and once every two years after that. He maintained good visual function and favorable ocular alignment at long-term follow-up (Figure 2).

**Figure 2:**
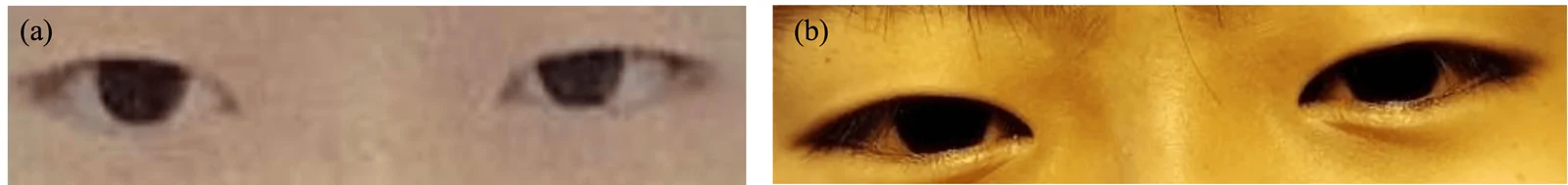
Images at 12 years and 1 month and 13 years and 3 months. (a) Photograph taken at 12 years and 1 month of age. (b) Photograph taken at 13 years and 3 months of age. Recovery of eye position was observed.

The chronological clinical findings, interventions, and visual outcomes are summarized in Table 1.

**Table 1:** Clinical findings and their management Prism diopters are denoted as Δ, esotropia as ET, and esophoria as EP.

Age		Findings	Response
Year	Month		
2	1	・Esotropia	・Prescribed spectacles (S+4.0 D in the right eye and S+4.5 D in the left eye)
	7	・Right aversion reaction ET' 40Δ	・Began right eye occlusion
3	3		・Prescribed spectacles (S+4.5 D, C+0.50 D, and Ax 90° in the right eye and S+5.0 D, C+1.0 D, and Ax 90° in the left eye)
	4	・Exacerbation of esotropia	・Prescribed a 20Δ base-out membrane prism on the right eye, and stopped occlusion of the right eye
4	3	・Scratch at the center of the spectacles	・Re-prescribed spectacles with the last power
5	4	・Visual acuity was (1.2×+4.50 D cyl+0.50 D Ax 90°) in the right eye and (1.2×+5.00 D cyl+1.00 D Ax 90°) in the left eye.・Eight out of nine questions answered correctly in the circles section of the Stereo Fly Test	
	7		・Prescribed spectacles (S+5.0 D, C+1.0 D, and Ax 90° in the right eye and S+5.0 D, C+2.0 D, and Ax 90° in the left eye)
	11	・Right eye: poor vision with membrane prism	・Prescribed 10Δ base-out membrane prisms on both eyes
6	0	・Right eye: poor vision after removal of membrane prism	・Began occlusion of the left eye
7	1	・Observed face turning to the left.	・ Planned surgical intervention
	4	・4ΔEP'/4ΔEP (both 10Δ base out membrane prism)	・Performed left lateral rectus resection (4 mm), postoperative strabismus angle showing 16ΔEP' and 14ΔEP.
	8	・16ΔEP'/14ΔEP without membrane prism	・Prescribed spectacles (S+4.5 D, C+1.0 D, and Ax 90° in the right eye and S+4.5 D, C+2.5 D, and Ax 90° in the left eye)
8	1	・Stereopsis: Incorrect response in the Fly section of the Stereo Fly Test ・Peering over spectacles observed	・Fitting adjustment resulted in four out of nine correct answers in the circles section of the Stereo Fly Test
9	5	・Peering over spectacles observed	・Prescribed spectacles (S+2.75 D, C+1.0 D, and Ax 90° in the right eye and S+3.0 D, C+2.5 D, and Ax 90° in the left eye)
10	10	・No correct answers in the animals section of the Stereo Fly Test	・Fitting adjustment resulted in four out of nine correct answers in the circles section of the Stereo Fly Test
11	7		・Prescribed spectacles (S+2.0 D, C+1.0 D, and Ax 90° in the right eye and S+2.5 D, C+2.5 D, and Ax 90° in the left eye)
12	1	・The spectacles were worn well, and all nine questions in the circle section of the Stereo Fly Test were answered correctly.	

## Discussion

One of the characteristics of this case is that the patient started treatment for accommodative esotropia but developed partially accommodative esotropia with strabismic amblyopia, yet ultimately achieved good binocular function.

On this basis, this case highlights several important points. First, the importance of early detection and intervention. Infantile esotropia can have a significant impact on visual development, especially if it develops in infancy, and there is a high risk that it will interfere with the acquisition of binocular function and lead to amblyopia [2,3]. In the present case, treatment was initiated earlier than 2 years and 1 month of age, which may have contributed to the favorable outcome.

Second, the importance of appropriate refractive correction. In accommodative esotropia, ocular position improves with an appropriate spectacle prescription but may occasionally shift to partially accommodative esotropia; therefore, ocular position needs to be assessed along with regular refraction examinations and reviews of the spectacle prescription [10]. In the present case, at the age of two years, ocular position improved only with correction of farsightedness, but at the age of three years, esotropia was not corrected by farsightedness correction alone and shifted to partially accommodative esotropia, requiring prisms and surgery to correct the ocular position.

The effectiveness of the occlusion treatment was also demonstrated. In this case, because the patient had developed partially accommodative esotropia, penalization with a membrane prism and closure of the healthy eye were performed, and visual acuity improved. In particular, the penalization effect of applying a membrane prism to the dominant eye was expected to promote the development of visual acuity in the amblyopic eye, and the alignment of the eye positions was believed to have contributed to the development of binocular function. Previous studies have demonstrated that prismatic correction in partially accommodative esotropia can maintain fusion and improve stereoacuity [8,11].

Regarding surgical treatment, stabilization of the eye position is necessary. Although there are reports that good binocular function can be achieved with early surgery, in this case, treatment of amblyopia was prioritized, and the visual axes of both eyes were aligned using a membrane prism [12]. However, due to the presence of face rotation, lateral rectus muscle resection surgery was performed at the age of seven years and four months, and good results were obtained.

Glasses were re-fitted once a year until the child was five years old, and then once every two years after that, and the prescription continued to be adjusted to accommodate changes in refraction. In this case, improvement of stereopsis was obtained by fitting adjustment of the spectacles. Notably, good stereopsis (140 seconds of arc or better) was consistently achieved when the residual esotropia was maintained at 4 Δ or less with optical correction, suggesting that fine motor alignment is critical for sustaining high-grade binocular function. It is also important to maintain the patient’s appropriate conditions of wearing glasses after periodic examinations.

As described above, in this case, the patient had a combination of accommodative esotropia, partially accommodative esotropia, and strabismic amblyopia, which were comprehensively treated by refractive correction, prism, surgery, penalization with a membrane prism, and occlusion of the normal eye, respectively. Therefore, it was necessary to confirm not only visual acuity but also binocular function and acquisition of stereopsis to evaluate the effects of treatment; however, some items could not be evaluated in young children, so long-term evaluation and intervention were necessary.

## Conclusions

We report a case of a child who initially presented with accommodative esotropia, subsequently developed partially accommodative esotropia with strabismic amblyopia, but ultimately achieved good binocular function, including high-grade stereopsis. In this patient, good visual function was maintained until at least the age of 12 years and 1 month through regular follow-up and adaptive therapeutic interventions. This case illustrates that the acquisition and maintenance of favorable visual outcomes in partially accommodative esotropia may be achievable through an appropriately tailored combination of refractive correction, prism therapy, amblyopia treatment, and surgical intervention, although the optimal timing and combination of these modalities may vary among individual patients.
